# Munificence of Dr. Nott

**Published:** 1857-03

**Authors:** 


### MUNIFICENCE OF DR. NOTT.

THE following statement from the *Albany Evening Journal*, in reference to a great and good man, is here given at length, as bearing directly on one of the main objects of this Journal, THE PRESERVATION OF THE HEALTH OF STUDENTS. It is a step forward in the right direction, for which the editor has spoken and written for nearly twenty years, to wit, the establishment of a professorship in colleges, for the express purpose of imparting instruction in reference to the preservation of the constitutions of the young. It carries with it more than ordinary weight, since the importance of it has forced itself on the mind of the president of a college, from the observations of half a century. It is within a day or two that I read a notice of the death of a young gentleman, and with a change of name only it would be an appropriate obituary of thousands of others. " He was the son of the pastor of the old church, who had educated his children, and they were noble men. He was a great favorite, and deservedly so. He was not well when he returned from college after graduating, and after a few weeks of struggling, he gave up entirely, and *lay down in his father's house to die.* It was a terrible blow to the father and the family." And well it was. How terrible, none but a father can ever know. And it is to prevent the reoccurrence of such incidents, by hundreds every year, that this publication is undertaken, and that Dr. Nott has founded a perpetual professorship in the last item but one of his princely donation:

" The following are the endowments. The several sums are to form a perpetual fund, the income only being used for the various purposes:

| | |
|---|---|
| For the establishment of nine Professorships $1,500 each per annum, . . . . . . . . . | $225,600 |
| Six Assistant Professorships or Tutorships, at $600 per annum, . . . . . . . . . . | 60,000 |
| Observatory, . . . . . . . . . . | 20,000 |
| Sixty-eight Auxiliary Scholarships, . . . . . | 50,000 |
| Fifty Prize Scholarships for under graduates, . . | 50,000 |
| Nine Prize Fellowships for graduates, $300 each, per annum, . . . . . . . . . . . . | 45,000 |
| Cemetery and Pleasure Grounds, . . . . . | 20,000 |
| Philosophical, Mathematical, and Chemical Apparatus, | 10,000 |
| Text Books, . . . . . . . . . . . | 5,000 |
| Scientific, Classical, Philosophical, Theological, Medical, and Law Books, . . . . . . . . . | 30,000 |

| | |
|---|---|
| Cabinet of Geological Specimens, . . . . . | 5,000 |
| Historical Medals, Coins, Maps, Paintings, and other Historical Memorials, . . . . . . . | 5,000 |
| Lectures on the Dangers and Duties of Youth, especially Students; the Development and Preservation of the Physical, Intellectual and Moral Constitution of Man; Preservation of Health, and on the Laws of Life, . . . . . . . . . . | 10,000 |
| To meet taxes, liens, assessments, incumbrances, insurance, and compensation to Visitors, and to make up any deficiencies in the income of any of preceding principal sums, so as to secure the attainment of the objects and purposes designed, . . . . . | 75,000 |
| Total, . . . . . . . . . . | $610,000 |

"There are to be five Visitors appointed, charged with the duty of acting in connection with the Trustees, and seeing that these trusts are faithfully carried out."

